# Integration Analysis of m^6^A-SNPs and eQTLs Associated With Sepsis Reveals Platelet Degranulation and *Staphylococcus aureus* Infection are Mediated by m^6^A mRNA Methylation

**DOI:** 10.3389/fgene.2020.00007

**Published:** 2020-02-26

**Authors:** Xuri Sun, Yishuang Dai, Guoliang Tan, Yuqi Liu, Neng Li

**Affiliations:** ^1^ Department of Critical Care Medicine, The Second Affiliated Hospital, Fujian Medical University, Quanzhou, China; ^2^ Respiratory Medicine Center of Fujian Province, Quanzhou, China; ^3^ Department of Outpatient operating room, The Second Affiliated Hospital, Fujian Medical University, Quanzhou, China; ^4^ Department of Pathogenic Biology, School of Basic Medical Sciences, Fujian Medical University, Fuzhou, China

**Keywords:** M^6^A, methylation, eQTL, sepsis, *Staphylococcus aureus*

## Abstract

Sepsis is a major threat with high mortality rate for critically ill patients. Response to pathogen infection by the host immune system is a key biological process involved in the onset and development of sepsis. Heterogeneous host genome variation, especially single nucleotide polymorphisms (SNPs), has long been suggested to contribute to differences in disease progression. However, the function of SNPs located in non-coding regions remains to be elucidated. Recently, m^6^A mRNA modification levels were revealed to differ at SNPs. As m^6^A is a crucial regulator of gene expression, these SNPs might control genes by changing the m^6^A level on mRNA. To investigate the potential role of m^6^A SNPs in sepsis, we integrated m^6^A-SNP and expression quantitative trait loci (eQTLs) data. Analysis revealed 15,720 m^6^A-cis-eQTLs and 381 m^6^A-trans-eQTLs associated with sepsis. We identified 1321 genes as locations of m^6^A-cis-eQTLs. These were enriched in platelet degranulation and *Staphylococcus aureus* infection pathways, which are vital for the pathophysiological process of sepsis. We conclude that m^6^A modification of mRNA plays a very important role in sepsis, with m^6^A-cis-eQTLs potentially having the most effect on individual variation in sepsis progression.

## Introduction

Sepsis is a serious systemic inflammatory response caused by bacterial, viral and fungal infection, and is one of the major causes of death in critical care patients (Rello et al., 2017). Data from developed countries estimate more than 30 million cases of sepsis and about 20 million cases of severe sepsis globally every year, which may result in more than 5 million deaths. Sepsis detection mainly depends on clinical diagnosis, but early clinical characteristics are not specific, and the lack of timely and reliable early warning diagnosis indicators is an important reason behind the high mortality among sepsis patients (Napolitano, 2018).

During the pathophysiological process of sepsis, pathogens and their toxins invade the vascular circulation, activate the host’s cellular immune system, generate cytokines and cause systemic inflammatory response syndrome (Rubio et al., 2019). Sepsis can affect various organs and systems of the body, causing necrosis and dysfunction of tissues and cells, and even multiple organ dysfunction syndrome (Ismail Hassan et al., 2020). Vascular endothelial cell damage, platelet function and immune dysfunction are key factors affecting disease progression (Khakpour et al., 2015; Guo and Rondina, 2019). Thus, resolving knowledge of pathogen invasion in sepsis will benefit diagnosis and treatment to reduce mortality of patients.

Treatment of sepsis is mostly aimed at pathogen control, including the early use of broad-spectrum antibiotics (Zhou et al., 2019). Removing the pathogen toxin is also very important. Lipopolysaccharide (LPS) (Gram-negative bacteria), lipoteichoic acid (LTA) (Gram-positive bacteria) and mannan (PLM) (fungi) all bind to toll-like receptors (TLRs) of innate immune cells (Yang et al., 2018). The lipid part of LPS can bind to TLR4 on many cells, LTA can bind to TLR2 and TLR6, and PLM can bind to TLR2, TLR4 and TLR6, activating the nuclear transcription factor-B (NF-B) signal pathway and the subsequent pro-inflammatory/anti-inflammatory cytokine response (Salomao et al., 2008). Blocking the pathogen toxin is therefore a critical step for preventing disease progression in sepsis patients.

Conversely, studies have shown that the susceptibility and prognosis of sepsis are genetically related to the host (Chapman and Hill, 2012; Rautanen et al., 2015). Heterogeneous host genome variation plays an important role in sepsis patients, suggesting another critical factor for diagnosis and treatment of sepsis. Genomic variation can produce gene expression differences among individuals. The location of expression quantitative trait loci (eQTLs) has been analyzed to resolve the relationship between sepsis and the host immune response and various signaling pathways (Davenport et al., 2016). However, most reported single nucleotide polymorphisms (SNPs) are in non-coding regions and have no obvious biological significance. The development of genetics and epigenetics research has revealed that some SNPs can influence RNA modification to control RNA secondary structure or regulate RNA protein interaction, modifying the function of enhancers, silencers and exon splicing (Ramaswami et al., 2015; Mao et al., 2016; Wu and Hurst, 2016). These studies show that some SNPs as well as eQTLs affect the stability and function of RNA, which is the basic mechanism of host genome variation associated with the disease.

Methylation of the sixth N atom on the adenine base is part of an important regulatory mechanism affecting RNA stability and functionality (Yue et al., 2015). In eukaryotes, m^6^A is the most common post-transcriptional modification of RNA; it can affect the specific recognition of a protein complex and RNA and thus affect the intracellular transport, shear processing, degradation and translation of mRNA, finally regulating gene expression (Dominissini et al., 2012; Alarcon et al., 2015). Key regulatory factors of m^6^A RNA modification, such as METTL3, METTL 14 and FTO, can control the occurrence and development of many diseases by regulating the level of m^6^A RNA modification (Li et al., 2017; Lang et al., 2019; Wang et al., 2019; Yue et al., 2019).

m^6^A-SNPs can be considered an important functional genetic variation, providing new clues for understanding the molecular mechanism of genetic variation. A large number of m^6^A-SNPs related to bone mineral density, periodontitis, rheumatoid arthritis and coronary heart disease have been identified through integration analysis of the m^6^A-SNP list (Mo et al., 2018a; Mo et al., 2018b; Mo et al., 2018c; Lin et al., 2020). They integrated GWAS data and m6A methylation to scope its role in disease. However, there is no report on the role of m^6^A modification in the development of sepsis. Identification of m^6^A-eQTLs associated with sepsis will extend our knowledge of the genetic mechanism of m^6^A modification in sepsis. We therefore used the open eQTLs data set to explore the potential role of m^6^A-eQTLs in the pathogenesis of sepsis.

## Materials and Methods

### List of m^6^A-SNPs

A list of m^6^A-SNPs was obtained from the m^6^Avar database (http://m6avar.renlab.org), with high (13,703 SNPs), medium (54,222) and low (245,076) confidence levels (Zheng et al., 2018). SNPs were obtained from dbSNP and TCGA. Other molecular interaction databases including starBase2 and CLIPdb were used during construction of this database.

### Sepsis-Related eQTLs

eQTL data were obtained through published articles (Davenport et al., 2016). In this study, 265 peripheral blood leukocyte samples from patients with sepsis were collected and their transcriptional profiles were detected. Genomic SNPs determining gene expression differences between cases were mapped as eQTLs, divided into cis- and trans-eQTLs. cis-eQTLs were restricted to 1 Mb regions between the SNP and associated probe. There were 644,390 SNPs associated with 17,347 mapping probes in sepsis patients.

### Identification of m^6^A-eQTLs in Sepsis and Gene Ontology (GO) Enrichment Analysis

m^6^A-SNPs obtained from the m6avar database were compared with previously described cis-eQTLs and trans-eQTLs to obtain m^6^A-cis-eQTLs and m^6^A-trans-eQTLs. A manffoman map was constructed according to p-values from statistical analysis of the original eQTLs. Since there were far fewer m^6^A-trans-eQTLs, corresponding genes were only analyzed for m^6^A-cis-eQTL locations. The number of m^6^A-cis-eQTLs possessed by these genes was determined. GO enrichment analysis was performed using the DAVID TOOL (https://david.ncifcrf.gov/summary.jsp) with the whole human genome as background. The GAD-DISEASE CLASS, GO biological process, GO molecular function, GO cellular component and KEGG pathways were used to classify these genes according their functional annotation.

## Results

### Sepsis-Related m^6^A-EQTLs Distribution Pattern

**Figure 1:**
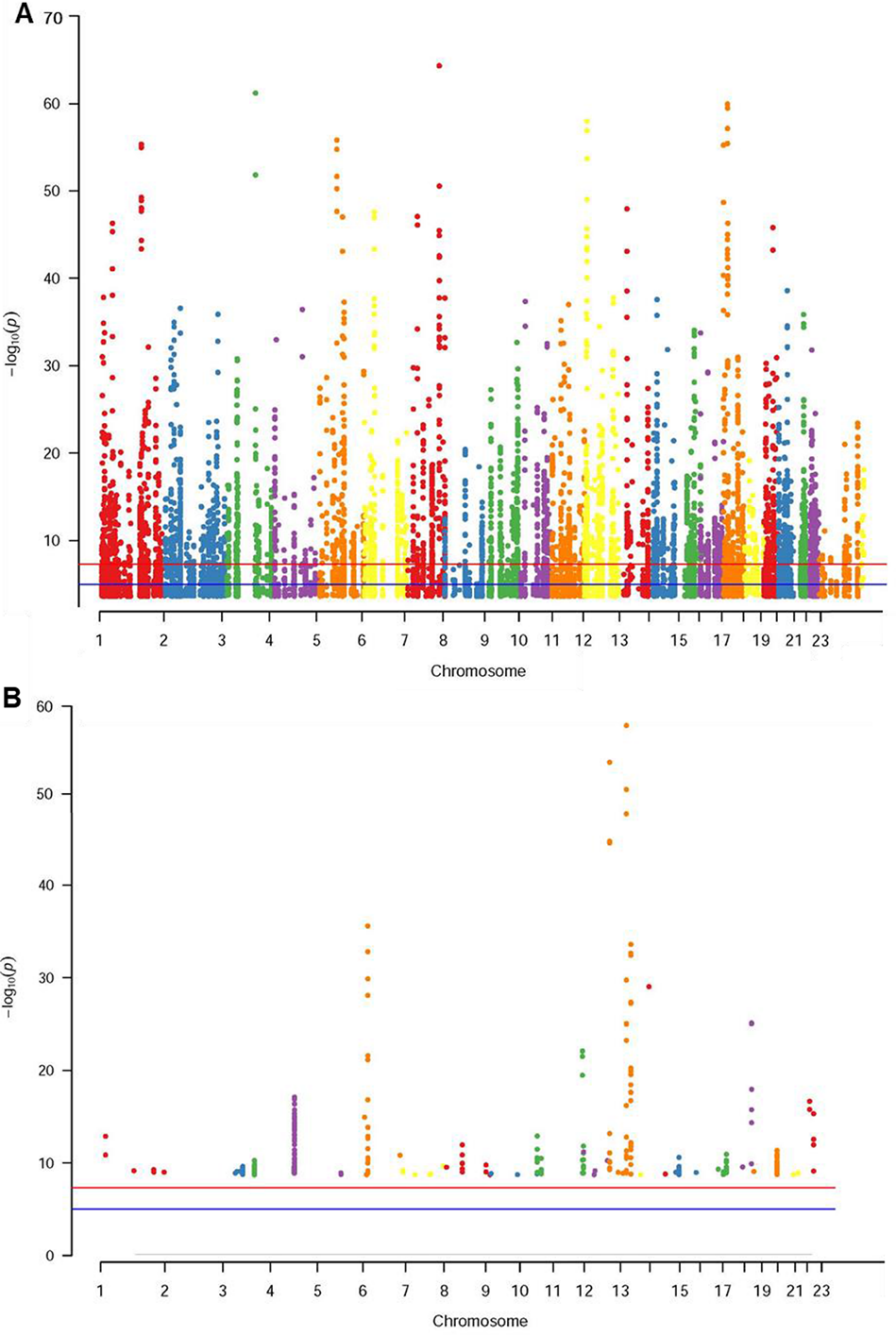
Manhattan plot of genome‐wide identified m^6^A-eQTLs in sepsis patients. The Manhattan plot displayed −log10 (p values) for each of m^6^A-eQTLs associated with sepsis. **(A)** m^6^A-cis-eQTLs **(B)** m^6^A-trans-eQTLs.

The database defined an SNP as one in which the typical sequence motif for m^6^A modification, such as DRACH, was changed (Zheng et al., 2018). High-confidence m^6^A-SNPs were identified from seven miCLIP and two PA-m^6^A-seq experiments, suggesting SNPs located close to the m^6^A site that may destroy the DRACH motif, such as D(A/G/U) to C, R(G/A) to C/T, A to C/G/U, C to G/A/U and H(A/C/U) to G. Medium-confidence m^6^A-SNPs were selected from 244 MeRIP-Seq experiments, which identify the intersection between SNP and m^6^A sites. Whether or not a SNP changes the DRACH motif or other characteristic sequences modified by m^6^A methylation was predicted by the random forest model. Low-confidence m^6^A-SNPs were predicted from the genome by random forest algorithms. We found 15,720 m^6^A-cis-eQTLs and 381 m^6^A-trans-eQTLs by integrated analysis of eQTLs associated with sepsis and more than 300,000 SNPs in the m^6^Avar database (**Figures 1A, B**, **Table S1**). According to the original sepsis eQTL study, a threshold of 1.00E-04 was used to test the association between m^6^A-eQTLs and gene expression in sepsis. As shown in **Figure 1A**, the p-values of m^6^A-cis-eQTLs ranged from 2.00E-04 to 5.19E-065, whereas p-values of m^6^A-trans-eQTLs ranged from 2.00E-09 to 5.93E-058. The m^6^A-cis-eQTLs were distributed on each chromosome with a similar pattern, displaying a gap close to the centromere. Among all m^6^A-cis-eQTLs, rs10239340 on chromosome 7 had the highest significance at p = 5.19E-065, followed by rs9849087 on chromosome 3 (p = 6.86E-062) (**Table 1**). Among the m^6^A-trans-eQTLs, rs10876864 on chromosome 12 had the highest significance at p = 5.93E-058, followed by rs11171739 on chromosome 12 (p = 1.56E-041) (**Table 2**).

**Table 1:** The top 20 most significant m^6^A-cis-eQTLs in sepsis.

SNP	Chr	Position	Gene	p value	FDR
rs10239340	7	1.29E+08	Interferon regulatory factor 5(IRF5)	5.19E-65	4.22E-58
rs9849087	3	1.21E+08	Golgin B1(GOLGB1)	6.86E-62	1.86E-55
rs8070859	17	15887789	Zinc finger SWIM-type containing 7(ZSWIM7)	1.22E-60	2.28E-54
rs9891938	17	15915072	Zinc finger SWIM-type containing 7(ZSWIM7)	3.52E-60	4.77E-54
rs7313235	12	10132283	C-type lectin domain family 12 member A(CLEC12A)	1.14E-58	1.16E-52
rs2285583	17	15968143	Zinc finger SWIM-type containing 7(ZSWIM7)	7.68E-58	6.93E-52
rs7313235	12	10132283	C-type lectin domain family 12 member A(CLEC12A)	1.31E-57	1.07E-51
rs1039320	5	73927752	Ectodermal-neural cortex 1(ENC1)	1.64E-56	1.02E-50
rs4792717	17	15948430	Zinc finger SWIM-type containing 7(ZSWIM7)	4.00E-56	2.17E-50
rs3785628	17	15970682	Zinc finger SWIM-type containing 7(ZSWIM7)	4.00E-56	2.17E-50
rs7522860	1	1.56E+08	Progestin and adipoQ receptor family member 6(PAQR6)	4.88E-56	2.48E-50
rs11150882	17	259648	Chromosome 17 open reading frame 97(C17orf97)	6.14E-56	2.93E-50
rs2025577	1	1.56E+08	Progestin and adipoQ receptor family member 6(PAQR6)	1.17E-55	5.30E-50
rs10474420	5	73934274	Ectodermal-neural cortex 1(ENC1)	1.84E-55	7.85E-50
rs7313235	12	10132283	C-type lectin domain family 12 member A(CLEC12A)	2.15E-54	8.73E-49
rs9884018	3	1.22E+08	Golgin B1(GOLGB1)	1.58E-52	5.85E-47
rs10942714	5	73922463	Ectodermal-neural cortex 1(ENC1)	2.32E-52	8.20E-47
rs10239340	7	1.29E+08	Interferon regulatory factor 5(IRF5)	2.93E-51	9.15E-46
rs6864196	5	73944444	Ectodermal-neural cortex 1(ENC1)	5.98E-51	1.80E-45
rs933489	1	1.56E+08	Progestin and adipoQ receptor family member 6(PAQR6)	5.72E-50	1.60E-44

**Table 2:** The top 20 most significant m^6^A-trans-eQTLs in sepsis.

SNP	chr	SNP position	Probe chr	Probe position	Gene	p value	FDR
rs10876864	12	56401085	17	8464669	LOC728823	5.93E-58	5.75E-48
rs11171739	12	56470625	17	8464669	LOC728823	4.82E-51	1.56E-41
rs705699	12	56384804	17	8464669	LOC728823	2.06E-48	5.00E-39
rs1384	12	69747834	5	43175395	Zinc finger protein 131(ZNF131)	2.56E-34	2.07E-25
rs10784774	12	69737879	5	43175395	Zinc finger protein 131(ZNF131)	2.31E-33	1.60E-24
rs2168029	12	69734641	5	43175395	Zinc finger protein 131(ZNF131)	3.90E-33	2.52E-24
rs6581889	12	69757429	5	43175395	Zinc finger protein 131(ZNF131)	6.04E-28	2.54E-19
rs10024529	4	1363886	19	41066160	Spectrin beta, non-erythrocytic 4(sptbn4)	7.88E-18	1.63E-09
rs1680032	4	1243573	19	41066160	Spectrin beta, non-erythrocytic 4(sptbn4)	8.09E-18	1.63E-09
rs1732115	4	1244416	19	41066160	Spectrin beta, non-erythrocytic 4(sptbn4)	8.09E-18	1.63E-09
rs1265923	4	1209174	19	44085725	LOC390940	1.40E-17	2.78E-09
rs1265923	4	1209174	19	41066160	Spectrin beta, non-erythrocytic 4(sptbn4)	4.23E-17	7.73E-09
rs730830	4	1240091	19	41066160	Spectrin beta, non-erythrocytic 4(sptbn4)	4.64E-17	8.06E-09
rs3817604	4	1291337	19	41066160	Spectrin beta, non-erythrocytic 4(sptbn4)	4.65E-17	8.06E-09
rs28429103	4	1320023	19	41066160	Spectrin beta, non-erythrocytic 4(sptbn4)	4.65E-17	8.06E-09
rs10024529	4	1363886	19	44085725	LOC390940	1.92E-16	3.11E-08
rs17164229	4	1078596	19	41066160	Spectrin beta, non-erythrocytic 4(sptbn4)	4.65E-16	7.40E-08
rs1721	21	46349496	15	75890708	Snurportin 1	4.91E-16	7.67E-08
rs1250116	4	1224587	19	41066160	Spectrin beta, non-erythrocytic 4(sptbn4)	9.58E-16	1.45E-07
rs884421	4	1227469	19	41066160	Spectrin beta, non-erythrocytic 4(sptbn4)	9.58E-16	1.45E-07

We then surveyed the relationship between eQTLs associated with sepsis and controllers of m^6^A modification such as writers, erasers and readers (METTL3, METTL14, WTAP, FTO, ALKBH5, YTHDC1, YTHDC2, YTHDF1, YTHDF2, YTHDF3) (**Table S2**). There was one eQTL in *YTHDF3* (rs7464, p = 2.42E-04), and 20 eQTLs in *YTHDC2* (p-values ranging from 2.64E-07 to 2.15E-04). Other m^6^A controllers were not associated with any sepsis-related eQTLs. However, none of the m^6^A controllers was identified as a significant gene for m^6^A-eQTLs.

### Sepsis-Related m^6^A-cis-eQTLs Locate to Thousands of Genes

To investigate the biological function of m^6^A associated with sepsis, we analyzed genes corresponding to m^6^A-cis-eQTLs. There were 1321 genes with an average of 11.8 m^6^A-cis-eQTLs, ranging from 1 to 195 (**Table S3**). There were 1038 genes with more than one m^6^A-cis-eQTL, and 52 genes had more than 50 m^6^A-cis-eQTLs. In particular, 10 genes had more than 100 m^6^A-cis-eQTLs: *RAD51C*, *LOC100129*, *LY6G5C*, *TRIM27*, *LOC642073*, *RFP*, *ABCC5*, *CLEC12A*, *WDR6* and *CAT* (**Table 3**). The most significant p-values for each m^6^A-cis-eQTL associated with sepsis ranged from 2.91E-012 to 1.14E-058. *CLEC12A* had 121 m^6^A-cis-eQTLs with highly significant p-values.

**Table 3:** The top 10 genes with the most number of m^6^A-cis-eQTLs in sepsis.

Gene	Freq	SNP	Chromosome	Position	p value	FDR
RAD51C	197	rs12935851	17	56600244	1.11E-31	2.19E-27
LOC100129668	190	rs2523685	6	31426256	3.39E-19	1.43E-15
LY6G5C	178	rs805290	6	31648403	6.27E-13	1.05E-09
TRIM27	157	rs3132377	6	28885974	6.91E-15	1.58E-11
LOC642073	155	rs6926737	6	32375745	6.75E-20	3.04E-16
RFP	145	rs6912843	6	28904162	2.91E-12	4.38E-09
ABCC5	128	rs7624838	3	1.84E+08	1.35E-13	2.54E-10
CLEC12A	121	rs7313235	12	10132283	1.14E-58	1.16E-52
WDR6	119	rs3212	3	49145741	6.25E-28	7.90E-24
CAT	100	rs11032695	11	34447586	7.35E-36	2.66E-31

### GO and KEGG Enrichment Analysis of m^6^A-cis-eQTLs Genes

**Figure 2:**
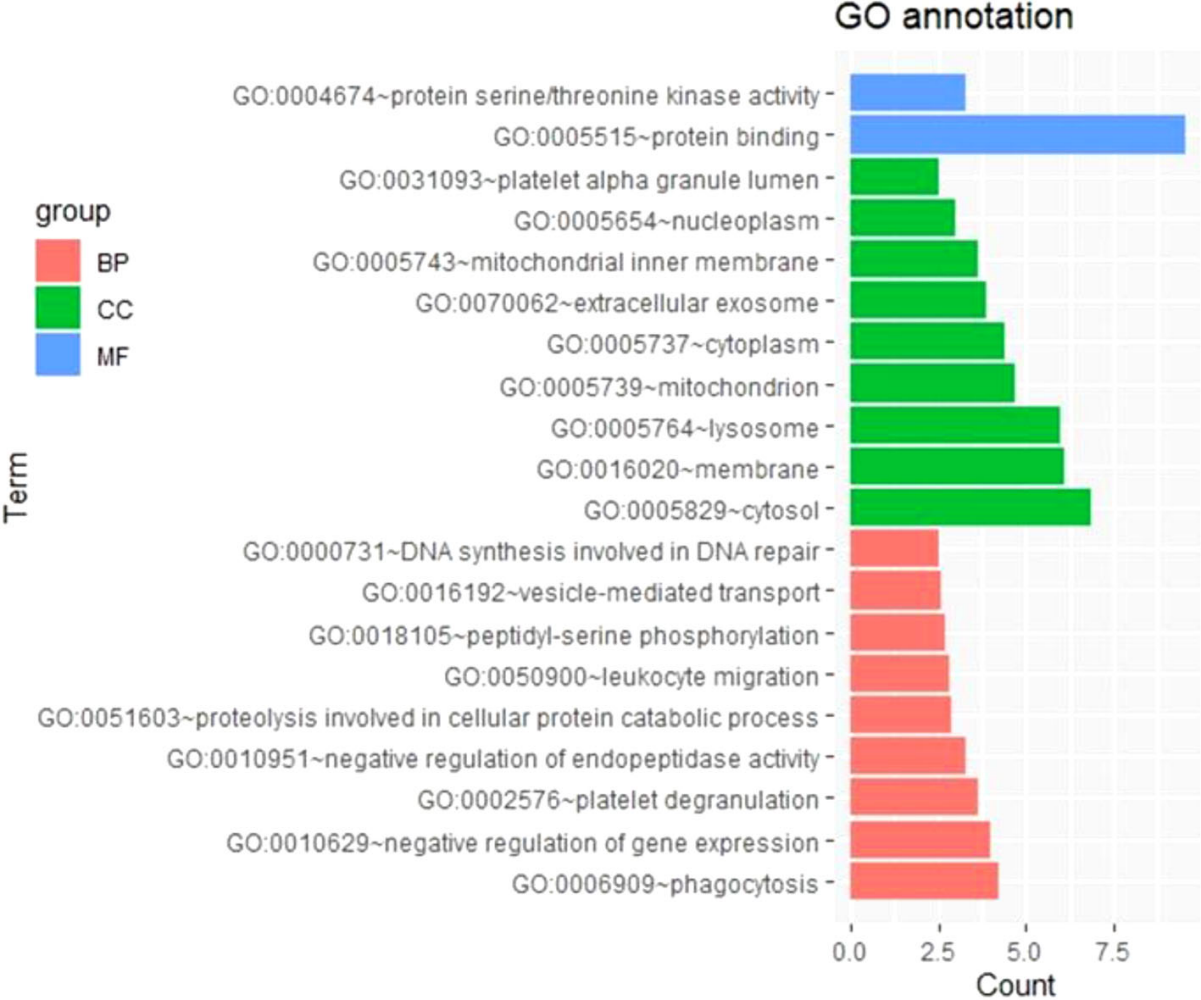
GO enrichment analysis displayed genes respond to m^6^A-cis-eQTLs are enriched in various biological processes (BP), molecular function (MF) and cellular component (CC). Note that platelet degranulation (BP) and platelet alpha granule lumen (CC) are significant pathway.

To further survey the biological function of genes linked to m^6^A-cis-eQTLs associated with sepsis, we performed GO enrichment analysis in a whole-genome background (**Figure 2**, **Table S3**). Among biological processes, genes containing m^6^A-cis-eQTLs were enriched in diverse pathways such as phagocytosis, negative regulation of gene expression, and platelet degranulation. Among cellular components, genes corresponding to m^6^A-cis-eQTLs were enriched in different compartments such as cytosol, membrane, lysosome and platelet alpha granule lumen.

**Figure 3:**
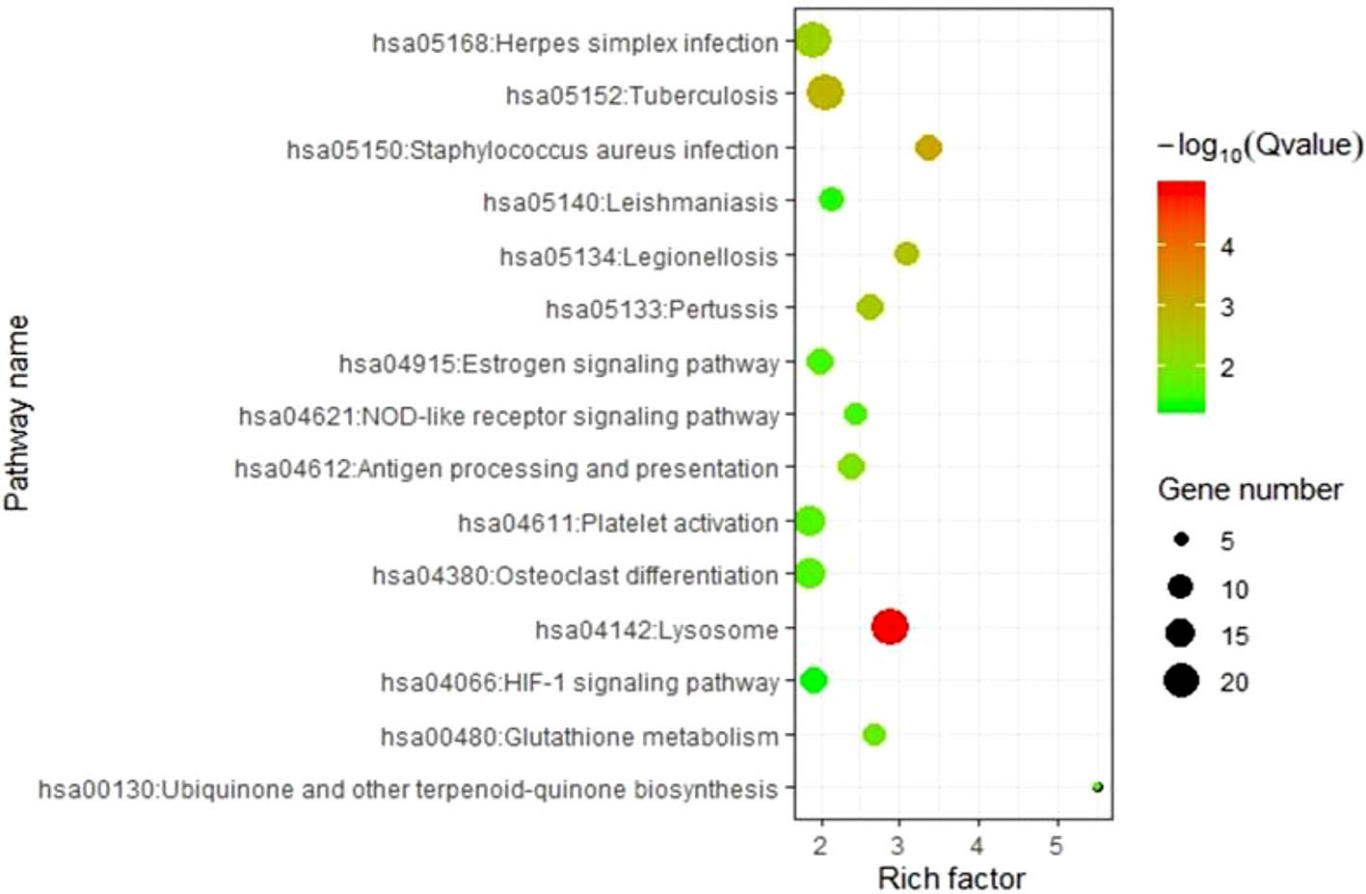
KEGG analysis revealed genes respond to m^6^A-cis-eQTLs are enriched diverse pathways. Note that *Staphylococcus aureus* infection pathway is with high significance.

To elucidate the molecular pathways involving genes corresponding to m^6^A-cis-eQTLs, we carried out KEGG pathway enrichment analysis (**Figure 3**, **Table S3**). Results revealed that these genes were enriched in multiple pathways such as Lysosome, *Staphylococcus aureus* infection, Tuberculosis, and Platelet activation. Among these, Lysosome was the most significant with p-value = 1.05E-05, followed by *S. aureus* infection with p-value = 6.44E-04.

To characterize the relationship between sepsis and genes corresponding to m^6^A-cis-eQTLs, we performed enrichment analysis using the disease database. Results showed that *TNF*, *HSPA1A*, *HSPA1B*, *TREM1*, *SOD2* and *MIF* genes related to sepsis correspond to m^6^A-cis-eQTLs.

### m^6^A-cis-eQTL Genes Related to Platelet Degranulation

We identified 17 genes related to platelet degranulation, *SERPING1*, *CD63*, *CTSW*, *TIMP1*, *CD9*, *VWF*, *ORM1*, *LAMP2*, *APP*, *CLEC3B*, *ITIH4*, *ABCC4*, *SERPINA1*, *CFD*, *QSOX1*, *ORM2* and *SRGN*, corresponding to m^6^A-cis-eQTLs associated with sepsis (**Table S3**).

### m^6^A-cis-eQTL Genes Related to *S. aureus* Infection

We identified 12 genes related to *S. aureus* infection, *C3AR1*, *FCGR2B*, *FCAR*, *C5*, *ITGB2*, *FPR2*, *C2*, *HLA-DPB1*, *CFD*, *HLA-DOB*, *ITGAM* and *PTAFR*, corresponding to m^6^A-cis-eQTLs associated with sepsis (**Table S3**).

## Discussion

Disease-related genes show genetic variation in the population, which can affect the occurrence and development of the disease including cancer, sepsis and infectious. For a long time, research on the functional mechanism of these genetic variations has focused on changes in biological functional activity of the encoded proteins. However, a large number of SNPs found in genome-wide association analysis do not correspond to functional regions of proteins. Similarly, eQTLs are not located in promoter regions regulating gene expression. In recent years, a large number of m^6^A methylation modifications have been identified on mRNA of eukaryotes (Yue et al., 2015). These regulate a series of biological processes by affecting the splicing, translocation, degradation and translation of mRNA involved in the occurrence and development of many diseases. Some SNPs that do not change the coded amino acid can regulate mRNA function by affecting the level of m^6^A modification, thus changing protein abundance. m^6^A-SNPs can change m^6^A modification levels to affect mRNA degradation rate; for example, change in mRNA secondary structure caused by altered m^6^A modification can affect mRNA binding with translation machinery. Similarly, m^6^A-SNPs can also affect mRNA transport from nucleus to cytoplasm. When m^6^A-SNPs are located at splicing sites, they can affect production of mature mRNA with correct biological function. Thus, m^6^A-SNPs can cumulatively regulate the function of key regulatory genes in the process of disease occurrence and development through multiple effects.

The role of m^6^A modification on mRNA in the pathogenesis of sepsis has not previously been reported. Therefore, study of m^6^A-eQTLs might aid in resolving the underlying mechanism of the pathogenesis of sepsis. By analyzing results of published research on eQTLs associated with sepsis and comparing them with overlapping m^6^A-SNPs in the m^6^AVAR database, we identified a large number of m^6^A-eQTLs related to sepsis, including 15,720 cis-eQTLs and 381 trans-eQTLs. As we expected, these m^6^A-eQTLs were distributed across all chromosomes, suggesting that m^6^A methylation of mRNA plays an important role in sepsis as in other diseases.

Among genes corresponding to m^6^A-eQTLs, we found some suggested as key players in the pathogenesis of sepsis. *HSPA1B*, with 61 m^6^A-cis-eQTLs, also named *HSP70*, is involved in the inflammatory response, which is an important biological step in the development of sepsis (**Table S2**). Research on different phases of sepsis found that HSPA1B can be considered a serum marker for the acute proinflammatory phase (Gasparotto et al., 2018). Study on Multiple organ dysfunction syndrome in sepsis found that increased HSPA1B has an anti-inflammatory effect (Wang et al., 2016). In rats, raloxifene prevents severe sepsis through induction of HSPA1B with an anti-inflammatory effect (Shen et al., 2017). Hesperidin can protect against lung injury induced by sepsis, also through induction of HSPA1B. Another protein, KNK43, also takes part in the HSPA1B pathway to coordinate the inflammatory response (Yuan et al., 2019). There is evidence that heat stress induces HSPA1B to prevent lethal sepsis (Matsathit et al., 2016). These results indicate that m^6^A-eQTLs exist in the key regulatory genes of sepsis and m^6^A methylation is closely involved in the process of sepsis, while genetic variation may cause a large number of individual differences through the mechanism of m^6^A methylation of mRNA.

We used GO enrichment analysis to characterize critical biological processes associated with an abundance of sepsis m^6^A-eQTLs. The platelet degranulation process was associated with 17 genes identified in this study. It is well accepted that platelet degranulation is a typical biomarker of sepsis. In the development of sepsis, platelet dysfunction plays a critical role in tissue injury (Assinger et al., 2019). Early onset neonatal sepsis can be predicted by platelet/lymphocyte ratio (Arcagok and Karabulut, 2019). A high level of platelet-derived growth factor B (PDGFB) is found in survivors of sepsis (Dou et al., 2019), and PDGFB can reduce the mortality of sepsis by blocking inflammatory responses (Dou et al., 2019). In sepsis-associated liver injury, AST/platelet ratio might be an early onset predictor (Dou et al., 2019). The underlying mechanism of platelet dysfunction in sepsis remains to be elucidated. Here, we revealed that genes corresponding to m^6^A-eQTLs are enriched in the platelet degranulation process, suggesting that m^6^A methylation is closely involved in the pathologies of sepsis. Published literature supports the relationship between the 17 platelet degranulation related genes corresponding to m^6^A-eQTLs and sepsis. ORM2 interacts with TLR2 signaling involved in the pathobiology of sepsis (Sumanth et al., 2019). CFD and VWF are important biomarkers of acute mortality for severe sepsis (Avriel et al., 2014; Gragnano et al., 2017). TIMP1 could be used as prognostic marker for the onset of sepsis (Nino et al., 2017). Sepsis is attenuated by inhibition of ABCC4 in rats (Xia et al., 2019). Reduction of CD63 increases the mortality rate of sepsis in mice through its function in the immune response (Yang et al., 2016; Alvarez-Estrada et al., 2019). The above evidence strongly supports our view that m^6^A methylation plays an extremely important role in the pathological basis of sepsis. We suggest that m^6^A-eQTLs may be a potential predictor of different clinical characteristics of the sepsis disease caused by genetic variation in individuals.

KEGG pathway enrichment analysis in this study revealed 12 genes corresponding to m^6^A-eQTLs associated with sepsis enriched in the *S. aureus* infection pathway. *S. aureus* is one of the major pathogenic bacteria in sepsis (Machado et al., 2019). Among the 12 genes corresponding to sepsis m^6^A-eQTLs in this pathway, platelet activating factor receptor (*PTAFR*) is induced in sepsis patients infected with Gram-negative bacteria (Dhainaut et al., 1994). Inhibition of PTAFR can block severe sepsis (Moreno et al., 2006). FPR2 is also induced in the early phase of sepsis and functions in cerebral inflammation (Gavins et al., 2012; Sordi et al., 2013). ITGB2 has been identified as an important factor in the inflammatory response to sepsis (Kragstrup et al., 2017). FCAR, a typical innate receptor for bacteria, is suggested to be an important protector against sepsis through mediation of bacterial phagocytosis (Chiamolera et al., 2001; de Tymowski et al., 2019). Such a high proportion of m^6^A-eQTLs related to *S. aureus* response genes indicates that m^6^A methylation plays an extremely important role in the occurrence and development of sepsis. The m^6^A-eQTLs we identified will be of value in the prognosis and diagnosis of sepsis.

In conclusion, we identified a large number of m^6^A-eQTLs potentially having critical functions in the pathogenesis and development of sepsis. These SNPs may contribute to the effect of genetic variation on the different outcomes of the disease. By mining the published literature, we found that these m^6^A-eQTLs are enriched in the platelet degranulation process and *S. aureus* infection pathway. Both are critical biological processes controlling the pathogenesis and development of sepsis, suggesting that mRNA m^6^A methylation plays a crucial role in sepsis.

## Data Availability Statement

All datasets generated for this study are included in the article/**Supplementary Material**.

## Author Contributions

XS and NL designed the project and wrote the manuscript. XS performed bioinformatics analysis. YD, GT and YL helped analysis of data and revised the manuscript.

## Conflict of Interest

The authors declare that the research was conducted in the absence of any commercial or financial relationships that could be construed as a potential conflict of interest.
